# The spread of the invasive species *Reynoutria japonica* Houtt. will both expand and contract with climate change: results of climate modelling for 14 European countries

**DOI:** 10.1002/ps.8732

**Published:** 2025-03-20

**Authors:** Nataliia Miroshnyk, Tetiana Grabovska, Hynek Roubík

**Affiliations:** ^1^ Institute for Evolutionary Ecology, National Academy of Sciences of Ukraine Kyiv Ukraine; ^2^ Research Institute for Sustainability – Helmholtz Centre Potsdam Potsdam Germany; ^3^ Department of General Ecology and Ecotrophology Bila Tserkva National Agrarian University Bila Tserkva Kyiv region Ukraine; ^4^ Department of Food System Sciences Research Institute of Organic Agriculture FiBL Frick Switzerland; ^5^ Department of Sustainable Technologies Czech University of Life Sciences Prague Prague Czechia

**Keywords:** biological invasions, biodiversity, alien plants, risk assessment, MaxEnt modeling, Japanese knotweed

## Abstract

**BACKGROUND:**

The study of invasive plant species distribution involves changes in their ranges and ecological niches under the projected global temperature increase until 2100. However, climate modeling of habitat suitability for *Reynoutria japonica* in Europe remains limited, hindering risk assessment and effective management of its spread. We used the MaxEnt model to assess the potential distribution of *R. japonica* in 14 European countries.

**RESULTS:**

It was found that the range of the taxon will expand into northern regions by 13.6% or 17.0%, depending on the scenario. However, range contraction in southern and central regions is expected to reach 26%. As a result, by 2100, a slight overall reduction in range (by 9–13%) is projected due to the decrease in distribution areas in southern parts of Europe, where maximum air temperatures will rise. Temperature variability throughout the year and precipitation during the warmest quarter are limiting factors for the spread. The minimum temperature of the growing season will influence distribution projections for 2060, whereas under current climate conditions, this parameter does not have a limiting effect. A general framework for controlling invasions of *Reynoutria* Houtt. taxa has been developed for both national and international levels.

**CONCLUSION:**

The study identified the dynamics of the invasive species' spread in Europe in relation to global climate change, assessed the risks of colonization in new areas, and provided tools for regulation and management to improve the prediction of potential distribution. © 2025 The Author(s). *Pest Management Science* published by John Wiley & Sons Ltd on behalf of Society of Chemical Industry.

## Graphical abstract

Technical scheme of the study. We conducted modelling (Maxent) and predicted changes in the range of *Reynoutria japonica* in Europe in response to climate change. This provided a theoretical foundation for the risk assessment and effective management of plant invasions.

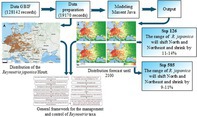

## 1 INTRODUCTION

Over the past 200 years, anthropogenic changes in land use have led to a decline in biodiversity, an acceleration of vegetation homogenization processes due to climate change, and significant spread of alien species. As biological invasions continue to expand and accelerate worldwide,^1^, ^2^, ^3^ climate change will further intensify these processes, particularly for species originating from southern and arid regions. This will lead to the spread and establishment of new weeds in agro‐landscapes and urban environments.

These changes will have a significant impact on food security, shift the focus of the chemical industry (herbicide production and other agricultural chemicals), and reshape green infrastructure in cities. The main factors driving the expansion of invasions include climate change, disruption of natural habitats, and the high competitiveness of introduced species in new environments. Their ability to form viable competitive hybrids and exhibit allelopathy, as seen in *Acer negundo* L., *Elodea nuttallii* (Planch.) H. St. John, and species of the *Reynoutria* genus, further facilitates their establishment and spread.

One of the main causes of global biodiversity loss is the spread of alien species.^2^, ^4^ The expansion of trade, transportation networks, the exploitation of waterways, and disasters on water routes (such as dam breaches and reservoir drawdowns) contribute to the introduction and spread of invasive alien species (IAS), posing a threat to biodiversity. EU Regulation 1143/2014 addresses the prevention of invasions and the management of IAS, requiring each member state to develop an action plan to prevent their unintentional introduction and spread.^5^, ^6^, ^7^

It has been found that an increase in mean annual air temperature in the future will lead to a nearly twofold rise in the number of alien species. Species originating from warmer regions of the world are the most susceptible to expansion due to climate change.^2^, ^8^ Therefore, understanding the potential distribution of invasive species in response to climate change, such as *Reynoutria japonica* Houtt. (= *Fallopia japonica* Houtt.), *Polygonaceae*, will help formulate effective management policies, regulatory measures, and ecosystem protection strategies against plant invasions.

*R. japonica* is a highly invasive species in the United Kingdom, continental Europe, North America, and parts of Oceania. It is listed among the 100 most invasive species in the world^6^, ^9^ and is rapidly expanding across Europe, driven by global climate change and the hybridization characteristics of species within this genus. The natural range of *R. japonica* includes the southern part of Sakhalin and the Kuril Islands, Japan, Korea, Vietnam, southwestern China, and Taiwan, where it occurs at elevations between 2800 and 3800 m above sea level. Within its native range, *R. japonica* thrives in various habitat types but prefers open areas, particularly along well‐lit mountain streams, riverbanks, roadsides, and forest edges. It is a pioneer plant capable of colonizing volcanic slopes. Currently recorded in 40 European countries, *R. japonica* poses a threat to global biodiversity, rapidly expanding and forming dense monocultural canopies that shade the ground. It produces a large amount of litter that decomposes slowly, as no specific soil microorganisms associated with this species have yet been identified.^6^, ^10^ The high hybridization ability of taxa within this genus has been well documented.^11^, ^12^ Studies^11^, ^13^, ^14^ indicate that *Reynoutria* taxa (particularly *R. sachalinensis* (F. Schmidt) Nakai and the hybrid *R. × bohemica*), are even more invasive and problematic due to their genetic variability, greater tolerance to extreme low temperatures and droughts, and, consequently, have unique ecological niche. This adaptability complicates risk management and control strategies for their spread under changing climate conditions. There is always a risk of species misidentification, particularly in databases such as the Global Biodiversity Information Facility (GBIF) and iNaturalist, and evidence suggests that the highly invasive hybrid *R. × bohemica* may be more widespread than current observations indicate.^12^

This issue is particularly critical for protected areas, as species and hybrids of *Reynoutria* sp. can irreversibly displace rare and endangered plant species.^14^, ^15^ Sexual reproduction and hybridization are key strategies for generating new genotypes and facilitating the spread of this genus, with the potential to produce highly productive genotypes. With climate warming, an explosive expansion of *Reynoutria* hybrids from south to north is expected.^12^ Authors have noted that hybrids closely related to *R. japonica* exhibited the highest rates of aggressive horizontal spread and competitor displacement within plant communities. Their ability to outcompete mycorrhizal tree species and accelerate nitrogen production by soil microbes contributed to their rapid and aggressive growth.^12^

The invasiveness of *Reynoutria* sp. has been confirmed at the landscape level due to the allelopathic effects of root‐secreted compounds. These compounds accumulate in soils with a history of invasion, restructuring microbial communities to their advantage, which potentially leads to increased biomass production through nitrogen accumulation.^14^, ^17^, ^18^ Root and/or rhizome exudates of *Reynoutria* sp., as well as plant residues, have been found to inhibit the growth of certain plant species, providing *Reynoutria* with a competitive advantage and disrupting the regeneration processes of native plants. Thus, the allelopathic properties of *Reynoutria* sp. may contribute to the expansion of their range.^19^ *Reynoutria* sp. taxa alter ecosystem interactions and reduce biodiversity by affecting plant diversity, aquatic fungi, and invertebrates in natural riparian habitats. They also disrupt food chains and other ecological connections, promote riverbank erosion by restructuring plant communities, and invade extensive riparian areas.^5^, ^11^, ^20^ Additionally, they hinder the regeneration of tree species in forests and reshape the structure of riparian forest stands.^14^, ^15^

Since comprehensive management and prevention strategies for invasions have not yet been developed, understanding the potential distribution of this taxon and assessing the risks of its establishment under climate change will help formulate effective surveillance, prevention, and monitoring programs. This knowledge will also contribute to understanding global trends in the spread of invasive species and the implementation of integrated, non‐chemical control strategies.^21^, ^22^ Species distribution modeling has proven to be an effective method for assessing potential spread based on presence data. In particular, the machine learning algorithm based on the maximum entropy model, MaxEnt, has demonstrated strong performance in this field.^14^, ^23^, ^24^

Thus, the objectives of this study are to (i) compare and model the potential distribution of *R. japonica* in 14 European countries using 19 bioclimatic variables from the WorldClim dataset and the MaxEnt model, (ii) identify key environmental factors that contribute to the spread of this species, which will indicate the susceptibility and sensitivity of ecosystems to invasions by *R. japonica* and other *Reynoutria* taxa, and (iii) propose a general framework for managing and controlling these invasive taxa.

The results of this study are crucial as a theoretical foundation for monitoring and managing invasions driven by climate change. They will help assess invasion pathways and risks in new territories, prioritize areas for invasion prevention, and identify already invaded regions requiring restoration and urgent management measures. Additionally, the study will support the implementation of environmentally safe management methods and enable the modeling of the species' response to future climate changes.

## 2 METHODS

**Figure 1:**
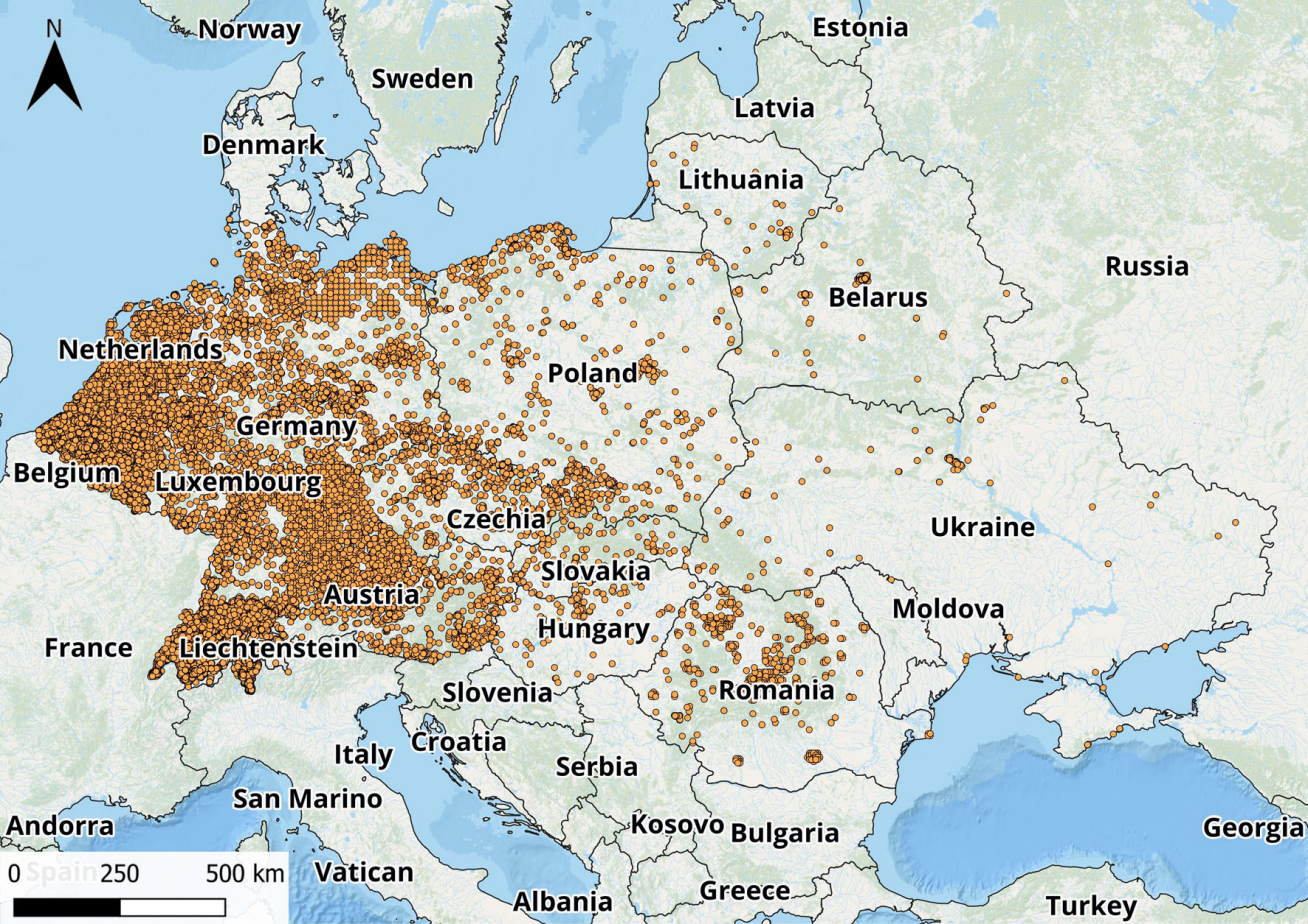
Locations of *R. japonica* according to the Global Biodiversity Information Facility (GBIF) database in 14 Europe countries.

The subject of the study is the spread of *R. japonica* across 14 European countries (Austria, Netherlands, Belgium, Belarus, Lithuania, Luxembourg, Germany, Switzerland, Czech Republic, Poland, Romania, Slovakia, Hungary, Ukraine). Field studies in Germany and Ukraine were conducted in the summer of 2023 using the transect method (Fig. 1 and Supporting Information, Table S1). The taxon *R. japonica* was identified using a combination of morphological features described by Bailey and Wisskirchen.^25^ Distribution data were obtained from the GBIF database^16^ and literature sources.^26^

Using recorded occurrences from the GBIF database^16^ and our personal findings in Germany and Ukraine, we modelled the spread of this species under different climate change scenarios up to 2100 using the maximum entropy approach^27^ in the MaxEnt 3.4.4 software package within the Java Runtime Environment.^28^, ^29^ MaxEnt can assess the contribution of climatic factors to the spatial distribution of biological species in three ways: through direct percentage contribution estimation, permutation importance assessment, and the jackknife method. The jackknife approach evaluates the contribution of individual variables to the final model.^29^, ^30^ Initially, all variables are excluded one by one except for the one being analyzed, and a model is built with the remaining variables. Then, a model is created using only a single variable (each in turn). Finally, a model is generated using all variables together for comparison. This method allows the assessment of how the removal of each variable affects model quality, highlighting the factors that play a key role in determining species distribution. During model training, MaxEnt tracks which environmental variables have the most significant influence on model construction.^27^

**Figure 2:**
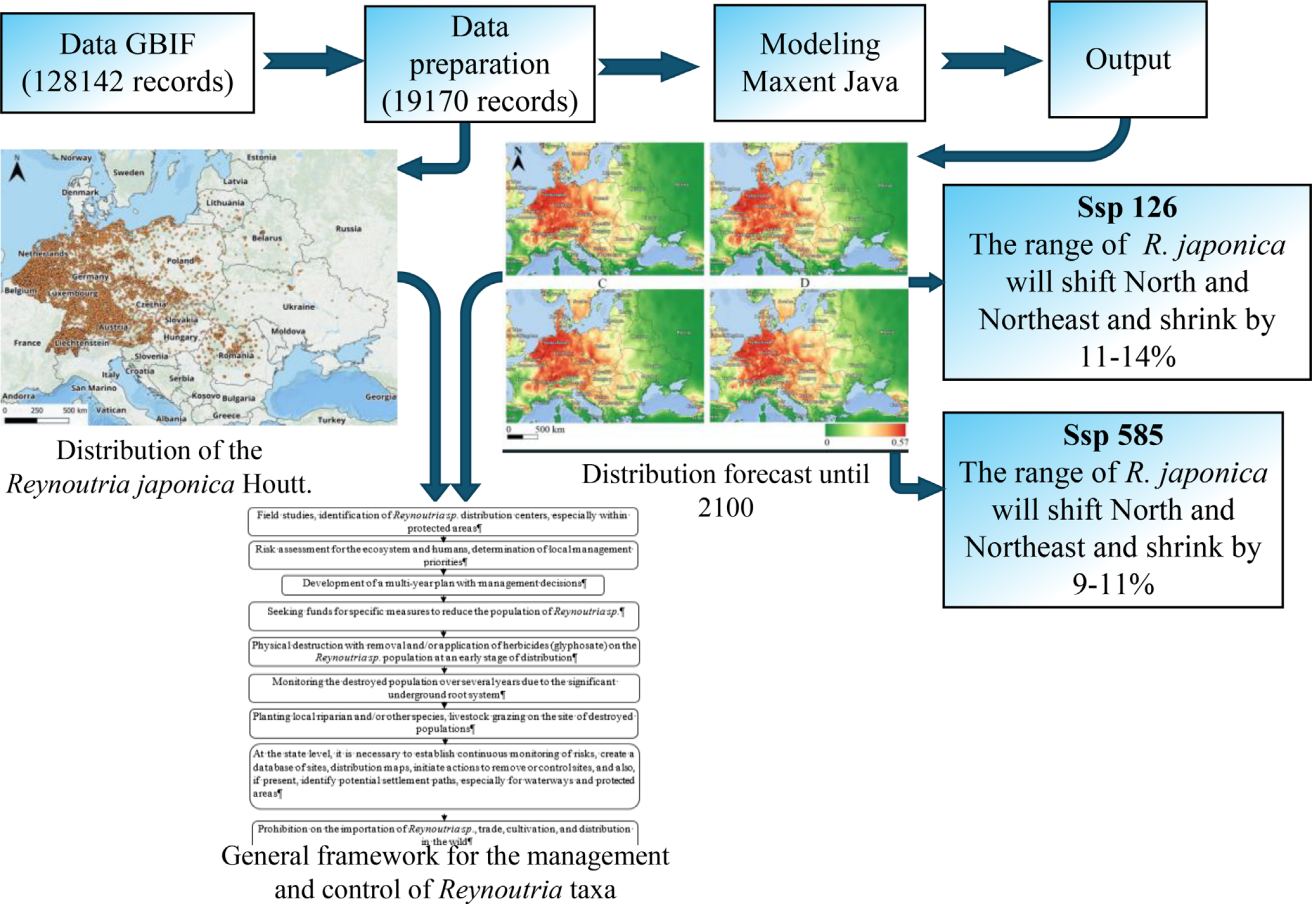
Technical scheme of the study.

The modelling process scheme, shown in Fig. 2, includes data acquisition from the GBIF database, literature sources, and our own field studies, followed by data cleaning to remove duplicates. It also involves obtaining environmental variable data, running the MaxEnt model, generating predictive maps for *R. japonica*, and calculating projected changes in its distribution area using QGIS 3.36.1, Diva‐GIS 7.5, and ArcGIS Pro 3.0.2.

We conducted modelling (Maxent) and predicted changes in the range of *Reynoutria japonica* in Europe in response to climate change. This provided a theoretical foundation for risk assessment and effective management of plant invasions (Fig. 2).

The bioclimatic data for the MaxEnt model were downloaded from worldclim.org and include 19 bioclimatic variables from WorldClim^31^, ^32^ with a resolution of 2.5 arc‐minutes per pixel, averaged over the period 1950–2000.^33^ For modeling future climate changes, climate data with a resolution of 30 s (~1 km^2^) were used (we used the GISS‐E2‐1‐G model) (Supporting Information, Table S2).

In our study, we utilized 128 142 records from the GBIF database^16^ (Fig. 1) documenting the occurrences of the species *R. japonica* across 14 European countries. Ultimately, 19 170 records were included in the analysis due to their high density per unit area, indicating proximity of locations and hence repeatability. Additionally, our 30 findings from 2023 were incorporated.

The parameter ‘Random test percentage’ was set so that the program randomly selected 25% of the data for further testing of the obtained model. Thus, 75% of the data was used for model creation as the training set and 25% as the testing set.^27^ The models were run with a default convergence threshold (10^−5^). To assess the forecast reliability of the model, the receiver operating characteristic (ROC) analysis method was used. The ROC curve is a visualization and evaluation tool for assessing the performance of a machine learning model capable of binary classification. The ROC curve is a way to see how well the model performs. The quality of the Maxent model was evaluated based on calculating the area under curve (AUC) of the ROC. The modelling quality can be interpreted as follows: 0.9–1 = excellent, 0.8–0.9 = good, 0.7–0.8 = acceptable, 0.6–0.7 = poor, and <0.6 = very poor (indicating unreliable modelling results).^34^ The logistic result of the model's prediction regarding environmental suitability for climate change scenarios was output in ArcGIS 10.1 (Esri, 2011), where 0 denotes unsuitability and 1 denotes suitability. The analysis of the significance of factors included in the model was conducted considering their percentage contribution to the training process for equation parameterization and the method of probability estimation of species occurrence by changing the ratios of indicators of one factor based on the baseline values of other indicators (permutation). The number of iterations was set to 500 to allow the model sufficient time for training.

To predict the impact of climate change on the potential distribution of the species during the periods 2041–2060 and 2081–2100, we used the GISS‐E2‐1‐G climate model,^35^, ^36^ developed by the Goddard Institute for Space Studies (GISS).^37^, ^38^ This model was employed to study climate changes based on various greenhouse gas emission pathways, land surface changes, and other factors under the ssp126 and ssp585 scenarios (Supporting Information, Table S3).

To assess changes in the distribution area of *R. japonica* under different climate scenarios, we utilized QGIS 3.6.1, ESRI ArcGIS Pro 3.0.2, and the Distribution Changes Between Binary SDMs tool from SDMtoolbox Pro v0.9.1.^39^ The distribution map based on GBIF data was modeled using QGIS 3.36.1, with the maps sourced from the ESRI map package for QGIS 3.36.1.

In Ukraine, quantitative assessment of the range expansion could not be conducted because the model excluded this territory from calculations due to insufficient data.

## 3 RESULTS

The density of *R. japonica* occurrences in the GBIF database varies across countries, with the following values (pcs/km^2^): Germany 0.05, Austria 0.024, the Netherlands 0.4122, Czechia 0.00896, and the lowest in Ukraine 0.00021, where the species is only at the early stages of expansion (Fig. 1).

The Maxent model for *R. japonica* is characterized by moderate classification performance in explaining the distribution of this taxon within the secondary range in Europe (AUC = 0.724 ± 0.003). With a substantial number of taxon findings included in the sample (19 170 occurrences), randomly split into training (75%) and testing (25%) datasets, the model evaluation criteria show very low variability (around 1% in relative terms) across four runs of cross‐validation. Visually, uncertainty regions are hardly noticeable (Supporting Information, Figs S2 and S3).

Omission for the test points correlates well with the predicted dynamics of omission. The AUC for our training and test data are almost identical at – 0.723 and 0.724, respectively, which is considered acceptable (Supporting Information, Fig. S1). The arithmetic mean value of 0.724 for AUC means a 72.4% likelihood that where species presence is predicted, it will actually be found (Supporting Information, Fig. S2).

The averaged distribution model results based on the ‘jackknife’ principle after four runs for *R. japonica* are presented in Table 1.

**Table 1:** Results for all locations modeled in the same Maxent run, including summary statistics and jackknife conclusions after four model runs

Species	Regularized training gain	Unregularized training gain	Iterations	Training AUC	Test samples	Test gain	Test AUC	AUC SD
*Reynoutria japonica* Houtt.	0.391	0.420	500	0.722	313	0.421	0.723	0.003

Abbreviations: AUC, area under the curve; SD, standard deviation.

### 3.1 The contribution of climatic factors to the forecast of the distribution of *R. japonica* species

Supporting Information, Table S4 shows the percentage contribution of each variable to the MaxEnt model, as well as the importance of their permutations for the model outcome.

Based on the results of four model runs (Supporting Information, Table S5), a hierarchy of the six most important variables influencing the distribution of *R. japonica* was identified: seasonality of temperature (BIO 4) > isothermality (BIO 3) > average temperature of the driest quarter (BIO 9) > average temperature of the hottest quarter (BIO 10) > precipitation of the hottest quarter (BIO 18) > annual temperature variation (BIO 7). These variables collectively accounted for 72.8% of the model's explanatory power. The most significant contribution to the model was from BIO 4 (temperature seasonality) (39.2%), which ranked second in importance (11.9%) among all variables. This indicates that temperature variability throughout the year strongly influences species distribution, meaning that large fluctuations in summer and winter temperatures currently act as limiting factors for the spread of *R. japonica*, including in Ukraine. The obtained data were confirmed after permutation testing, as the values remained high and the ranking order was nearly unchanged (Supporting Information, Table S5). BIO 3 (isothermality) was the second most significant variable, contributing 22.4% with a high permutation importance of 14.5%.

BIO 9 (mean temperature of the driest quarter) contributed 21.9% to the model, but after permutation and four model runs, this dropped to 9.1%, ranking third in importance. Low temperature during the dry period can limit resource availability for the species. This can be a crucial factor in understanding how the species adapts to dry period conditions.

BIO 10 (mean temperature of the warmest quarter) had a low contribution to the model at 5.0%, with an importance of 4.8%, placing it fourth in importance. BIO 18 (precipitation of the warmest quarter) had a small contribution (4.4%), but the highest importance (27.7%) among all variables. This indicates that the amount of precipitation during the hottest period of the year is a critical factor for the species' survival.

BIO 11 (mean temperature of the coldest quarter) had a low contribution to the model at 2.2%, but an importance of 7.4%, higher than BIO 10. All other bioclimatic factors had almost no impact on the model construction and hence on the species distribution.

After four runs, the contribution to the model of BIO 7 (temperature annual range) had decreased to 0.7%, dropping to tenth position, but its significance slightly increased (to 4.8%).

The bioclimatic variable with the greatest gain when used in isolation was BIO 3 (isothermality), which thus carries the most important information by itself (Supporting information, Fig. S3).

The results of the Jackknife technique applied to analyze the importance of climatic variables for the distribution of *R. japonica* (Supporting information, Fig. S3, test data) showed that the most important variables for the species distribution were BIO 3, BIO 4, BIO 9, and BIO 7 (temperature annual range). When evaluating the model using Jackknife, the greatest gain was provided by the parameter BIO 3. In the analysis with variable exclusion, BIO 3 contains unique information, and its exclusion leads to significant model deterioration. Therefore, the parameters BIO 3 (isothermality), BIO 18 (precipitation of the warmest quarter), and BIO 11 (mean temperature of the coldest quarter) are important for predicting future changes in species distribution, although they do not currently make significant corrections to the model construction.

**Figure 3:**
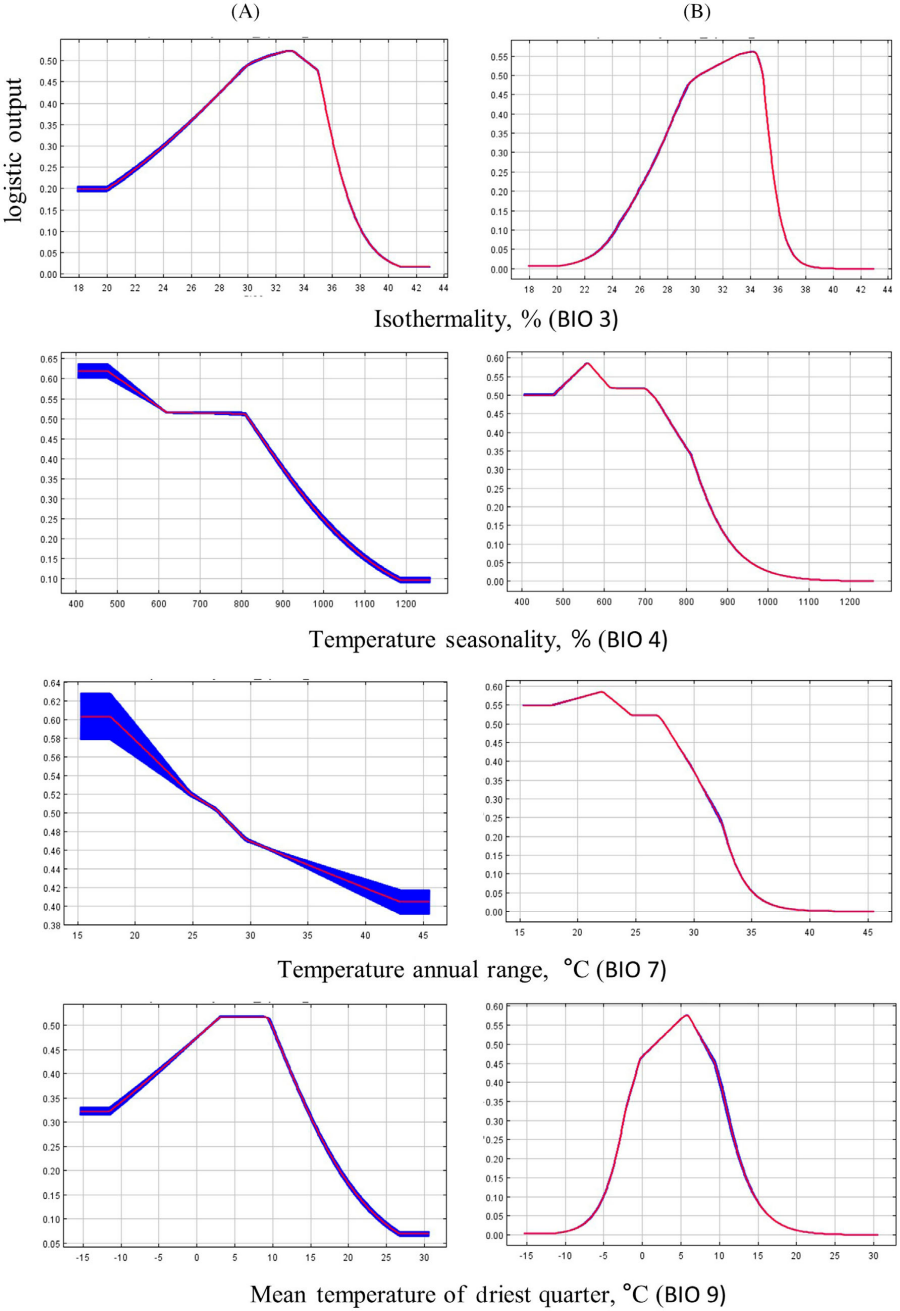
Predictive curves (*complementary log–log function*) of the most important climatic indicators limiting the distribution of *R. japonica* (ecological niche). (A) All factors are considered in the model as independent variables. (B) Only one significant factor is considered in the model.

The response curves (probability of species occurrence) calculated based on the values of the independent variables included in the model (Fig. 2) characterize the optimal and limiting values of environmental factors (effectively the ecological niche) for *R. japonica*. The most significant factor in the model for species distribution is BIO 3 (isothermality), a synthetic indicator that considers annual temperature fluctuations (BIO 1) and extremes, the minimum and maximum temperatures of the coldest and warmest months, respectively. As BIO 1 and temperature extremes increase, BIO 3 will also increase. Modeled climate changes will result in increased values of BIO 1 and extreme indicators, leading to a shift in the ecological niche boundaries of *R. japonica* by 17–26% under a negative climate scenario ssp 585 and a decrease in the importance of minimum temperature extremes for its distribution (Fig. 3 and Supporting information, Fig. S3, BIO 3). The values on the graph correspond to the highest response around 30–35%, indicating that the species prefers conditions with moderate temperature fluctuations throughout the year.

The second most significant factor is BIO 4 (temperature seasonality), which indicates changes in temperature throughout the year. Large fluctuations can affect growth cycles as well as competition with other species. A sharp decrease in values at a coefficient of variation of 0.5 indicates the importance of stability and moderate seasonal temperatures for the distribution of *R. japonica*.

The mean annual temperature range (BIO 7) is the third most significant factor and can be useful in studying the impact of extreme temperatures on species distribution. The peak value range lies around 15–20 °C, indicating relatively small temperature fluctuations (Fig. 3, BIO 7). The mean temperature of the driest quarter (BIO 9) reaches peak probability at 6 °C and maintains a stable plateau up to +15 °C, after which there is a sharp decline on both sides of the curve. For the distribution of *R. japonica*, non‐extreme summer temperatures are important, meaning that both temperature extremes – the minimum and maximum – are significant, especially when moisture is lacking.

**Figure 4:**
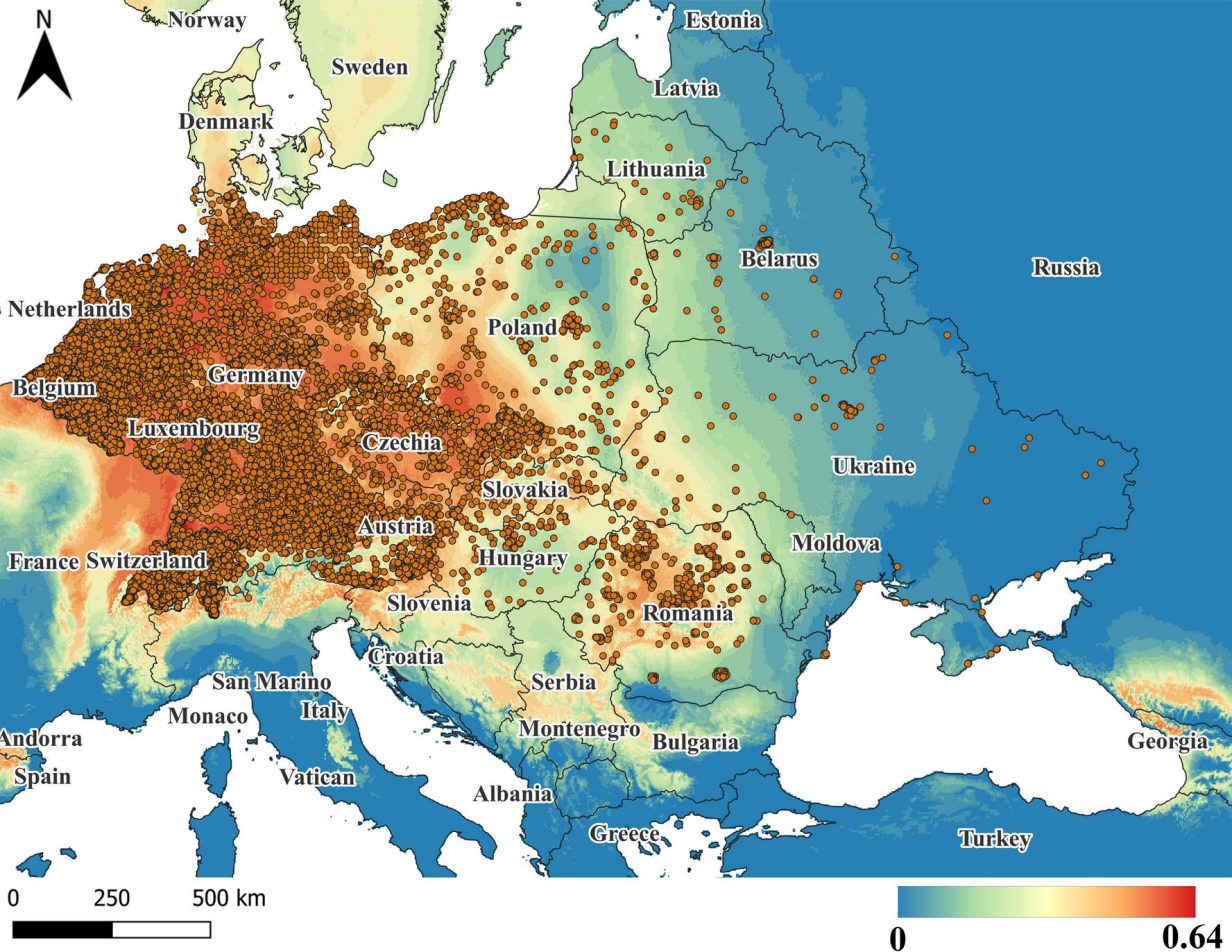
Current and forecast of probable distribution of *R. japonica* as of 2023. Blue corresponds to minimal probability of species distribution and red to maximum, where the range 0–0.64 represents the coefficient of probability of finding the species at specific points.

When analyzing the map of the potential and current distribution of *R. japonica*, created using all data in the MaxEnt program (Fig. 4), it was found that with the highest probability (species presence coefficient of variation 0.64), populations will spread to northern Ukraine, Belarus, and Lithuania.

In the forecast of the impact of future climate change under the optimal scenario ssp126 for the years 2041–2060 on the distribution of *R. japonica*, it has been identified that the climatic factor BIO 11 (mean temperature of the coldest quarter) is crucial for current distribution (Supporting information, Fig. S3, test gain). In the forecast, this factor will become increasingly significant over time.

**Figure 5:**
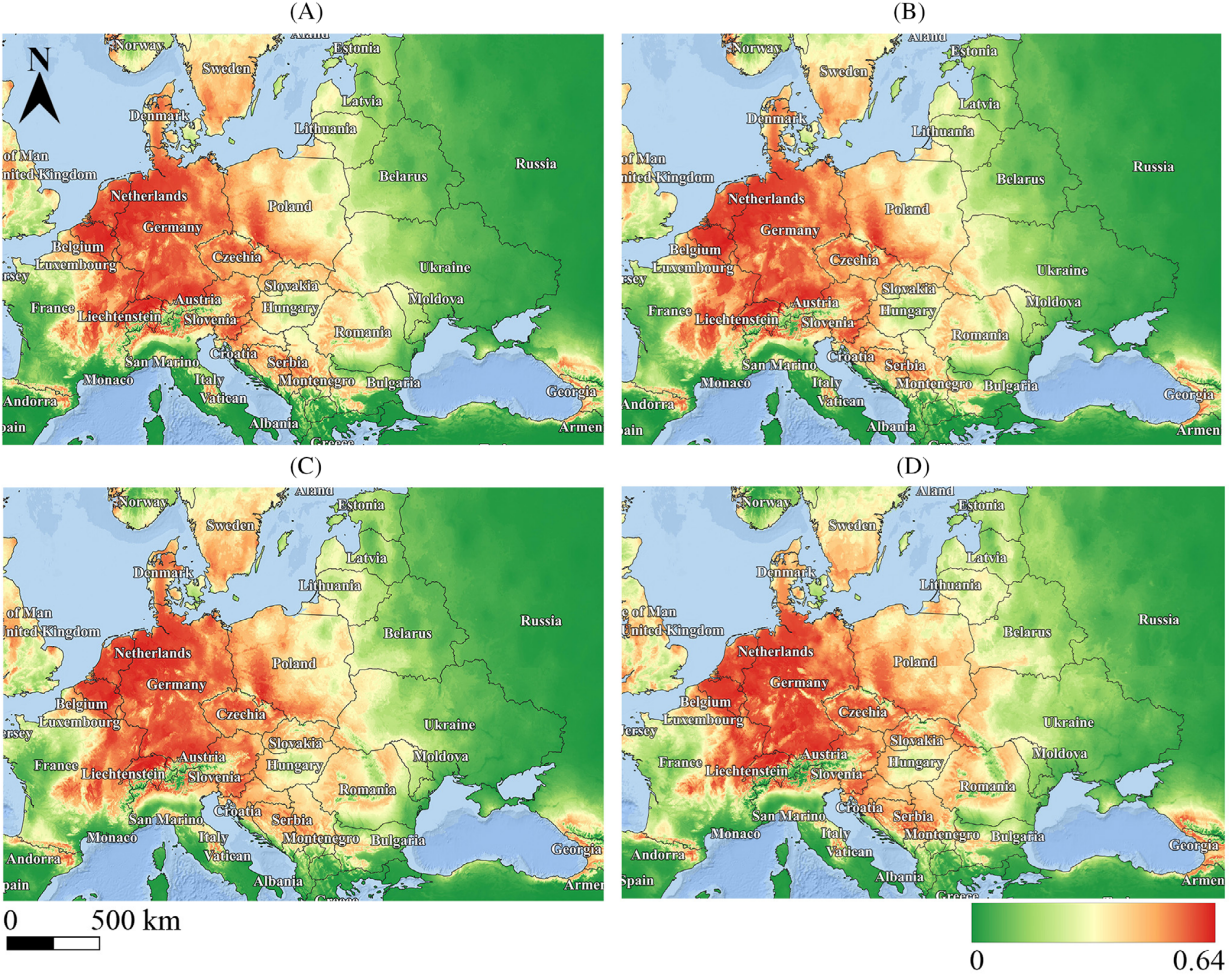
Map of the distribution of *R. japonica* across 14 European countries considering global warming using the GISS‐E2‐1‐G climate model: (A) ssp126, 2041–2060; (B) ssp126, 2081–2100; (C) ssp585, 2041–2060; (D) ssp585, 2081–2100.

The projected future distribution of the species under two climate change scenarios is depicted in Fig. 5.

The AUC values for all four forecasts, ranging from optimistic to pessimistic scenarios for the periods 2041–2060 and 2081–2100, vary within the range 0.636–0.640, indicating a moderately predictive capability of the model. For more accurate conclusions, additional analysis is necessary, including checking other metrics and assessing the quality of data used to train the model. Overall, with increasing global warming, areas with a moderate climate pose potential risks for the spread of *R. japonica*.

**Figure 6:**
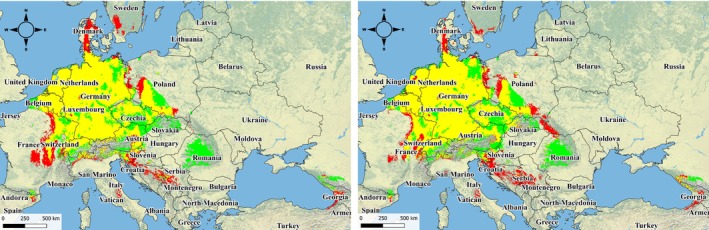
Map of *R. japonica* population distribution according to projected climate changes (left map ‐ ssp126 (2081‐2100 рр.) – 2,97 °C; right map ‐ ssp585 (2081‐2100 рр.) – 5,62 °C): red – area where populations will expand considering increased temperatures from global warming; yellow – area where the distribution of *R. japonica* will remain unchanged under both climate scenarios; green – area where populations are currently distributed and where they will be absent by 2100 according to projected scenarios due to global warming.

We evaluated changes in the area of distribution of *R. japonica* under different climate scenarios (Fig. 6). It was found that there will be an expansion of the range by 16.3% by 2060 under the pessimistic scenario and up to 17.0% by 2100. However, in areas where the species is most prevalent, there will be a reduction in range due to increased maximum temperatures in summer, up to 27.4% by 2060 under the pessimistic scenario. Therefore, the projected net reduction in the southern range, excluding expansion in the north, amounts to 14% for the optimistic scenario and 10% for the pessimistic scenario.

Thus, in the future with climate change, *R. japonica*'s range will shift northward and north‐eastward and contract by 11–14% under the optimistic ssp126 scenario or by 9–11% under the pessimistic ssp585 scenario (Fig. 6).

The green areas in Fig. 6 can be explained by the fact that with global climate change and rising air temperatures, there will be a reshaping of biodiversity distribution ranges as limiting factors and ecological niches change. For example, modeling for *R. japonica* indicates changes in moisture levels and an increase in the average temperature of the warmest quarter, which will lead to a contraction of its range and ecological niche in central Europe and an expansion northward and westward (Fig. 6, optimistic scenario ssp126) or northward, westward, and eastward (Fig. 6, pessimistic scenario ssp585).

A distinctive feature of its southern distribution is the forecasted colonization of mountainous regions (upward along altitude gradients), as within its natural range *R. japonica* ascends to 3800 m above sea level. Further research is needed to explore these characteristics.

## 4 DISCUSSION

Populations of the invasive species *R. japonica* pose a threat to global biodiversity because they displace native species, restructure ecosystems, and cause significant economic losses.^6^, ^8^, ^14^, ^40^, ^41^, ^42^ The greatest threats associated with the widespread expansion of *Reynoutria* taxa include genetic plasticity (formation of viable hybrids with higher invasiveness than the parental taxa), allelopathy, high vegetative productivity, the ability to colonize habitats along both altitudinal and latitudinal gradients, and tolerance to a wide range of moisture conditions. This species can invade both submerged plant habitats (*hydrophytes*, aquatic plants) and dry urbanized environments.

We conducted climate modeling of the range of *R. japonica* in 14 European countries under two climate change scenarios, which is crucial for biodiversity conservation and for making effective decisions on risk assessment and preventing the spread of this taxon.

The limiting factors for the distribution of *R. japonica* are the lowest temperatures of the cold season and the highest temperatures of the warmest season, which are expected to diminish and shift with ongoing global warming. Given its plasticity to various climatic conditions, high adaptive potential, intensive vegetative reproduction, and the formation of highly adapted and viable hybrids, it can be assumed that further global climate change will facilitate the broader and faster spread of this taxon, particularly into areas that are currently less favorable due to excessively low cold‐season temperatures.

For southeastern Europe, the most influential environmental factors regarding the invasion of *Reynoutria* sp. were identified as follows: (1) precipitation in the driest quarter of the year, (2) precipitation in the warmest quarter, (3) average temperature of the driest quarter, and (4) average temperature of the coldest quarter of the year. Models in this study demonstrated a dependence of spread on water regime.^14^ The findings^14^ align with the conclusions of Bailey and Wisskirchen^25^ regarding the constraints these taxa are limited by due to consistent water supply throughout the year, as well as with observations on the dependence of *R. japonica* existence on the sum of heat and minimum temperature during the growing season.^43^

While in our study the most significant factor influencing the distribution of the taxon is temperature variability throughout the year (seasonality), driven by substantial temperature differences between summer (up to +36–38 °C) and winter (down to −30°C or lower in some years), other important factors include the mean temperature of the driest quarter, isothermality, mean temperature of the warmest quarter, and precipitation in the warmest quarter. In particular, temperature variability throughout the year and precipitation in the warmest quarter act as limiting factors for distribution. The minimum temperature of the growing season is expected to impact the species' range in projections up to 2060, whereas under current climate conditions, this parameter does not have a limiting effect. Therefore, our modeling results differ significantly from those reported^14^ for European countries. This discrepancy can be attributed to the larger number of occurrence records in our study area and differences in model parameter adjustment methods.

The authors declared that the southern boundaries of *R. japonica* growth will shift northward in response to intensified summer droughts.^43^ The results for Europe and our data for Germany and Ukraine confirm this.^14^ The authors indicate that according to climatic modeling for *R. japonica* and *R. × bohemica* in southeastern Europe and along the rivers of Slovenia and Croatia, expansion of their range in coastal habitats is forecasted to increase by 30–40%, with broader distribution across southeastern Europe over the next 25 years.^14^ Climate change could facilitate an expansion of *R. japonica*'s range in coastal habitats by 32.4%. In our modeling results, expansion into northern regions for the positive scenario amounts to 13.6% and for the negative scenario amounts to 17.0%. However, range contraction in southern regions due to increased temperatures is projected to reach 26.5% for both scenarios, with a real projected reduction in range of 9–13% by 2100.

A severe invasion into the rivers of Slovenia and Romania requires urgent preventive measures, as most *R. japonica* populations in these countries are expected to remain stable.^14^ Our research indicates significant expansion in Germany, particularly into ruderal and urbanized habitats.

*R. japonica* has broad temperature tolerance in the southern regions of Sweden, enduring temperatures as low as −30 °C.^43^ In southeastern Europe, this species can withstand −4.2 °C during the coldest month of the year under current climatic conditions.^14^ Furthermore, according to future climate projections, a minimum temperature of −4.75 °C in the coldest month will still be suitable for its survival. Therefore, with ongoing climate warming and increasingly mild winters, the spread of this taxon is expected to accelerate. This is supported by its significant distribution in Germany, where the average temperature of the coldest month is −2.8 °C, while the minimum recorded temperature for the coldest month is −45.9 °C.^33^

The parameter ‘annual precipitation sum greater than 500 mm’ (annual PPT) may be more significant for the distribution of *R. japonica* near the eastern boundary of its range than low extreme temperatures.^43^ However, modeling data for southeastern Europe suggest that the mean temperature of the coldest quarter and precipitation in the warmest quarter may have the greatest influence on the spread of *R. japonica* populations in Serbia and adjacent areas.^14^ According to our results, the limiting parameter that will become increasingly important with climate change is precipitation in the warmest quarter, along with temperature variability throughout the year.

Thus, global climate change will not uniformly affect distribution patterns. Increases in overall temperature and temperature extremes may lead to significant range contractions, while other species may acquire invasive properties. *R. japonica* exerts a complex and negative transformative impact on biodiversity at both ecosystem and landscape levels due to its high invasiveness, genotype plasticity, and allelopathy. Urgent management actions are required at a pan‐European level, particularly a comprehensive approach integrating experimental bioregulatory methods, which may prove most effective but also entail risks of biosecurity (such as explosive proliferation of non‐European but natural pests for *Reynoutria* sp., ecosystems restructuring through food chain disruptions, etc.).

### 4.1 Risk management of the spread of populations of the genus *Reynoutria*

The regulation and management of *Reynoutria* taxa distribution are implemented in many European countries and involve significant economic costs.^7^ For example, the municipality of Amsterdam allocated €8.2 million for eradication efforts in 2019.^8^, ^40^ At the international level, substantial funds have been allocated for the development of biological control methods to combat the spread of this taxon.^41^, ^44^ Currently, national and international legislation on risk control and management of alien species distribution is being developed and refined, such as EU Regulation 1143/2014.^45^

**Figure 7:**
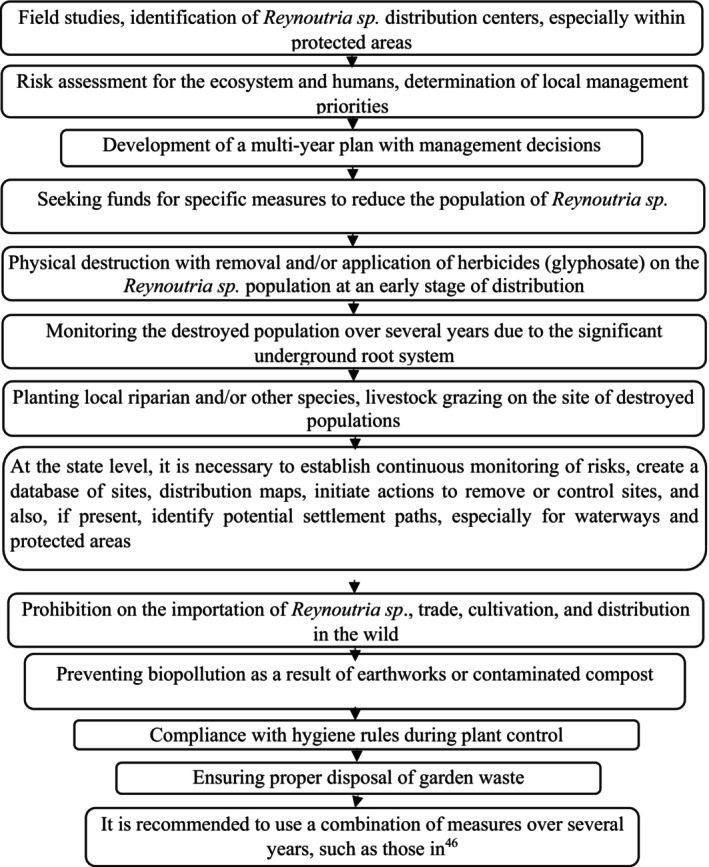
General framework for the management and control of *Reynoutria* taxa invasions.

We have developed a general framework for the management and control of *Reynoutria* sp. distribution (Fig. 7). Additionally, widespread media coverage and educational efforts are necessary, as we have observed significant invasions in Germany, Croatia, Hungary, and Ukraine within public institution areas, including kindergartens, universities, hospitals, zoos, flowerbeds, and residential areas. The species is also prevalent in urban green spaces or abandoned lots, such as near large parking areas, highways, and shopping centers, where plants are left to grow and flower. This presents a direct risk for further hybridization and increased invasiveness. Managing *Reynoutria* sp. populations is particularly crucial, as preventing generative reproduction is essential to avoid the emergence of increasingly resilient, fertile, and genetically diverse hybrid generations.

For effective ecosystem and landscape management, it is necessary to assess invasion risks and develop management strategies at both national and international levels to reduce the spread of invasive alien species. The proposed *Reynoutria* invasion control framework can be used to establish continuous risk monitoring, create databases of occurrence records and distribution maps, implement eradication or control measures in areas where the species is already present, and identify potential dispersal pathways, particularly for aquatic corridors and protected areas (Fig. 7). To enhance management efforts, the results of our mapping and predictions can be utilized for database development, spatial and temporal distribution mapping, climate‐based modeling of alien species spread, and the study of all possible invasion pathways of invasive species.

Current climate modelling has proven effective in evaluating the risks and consequences of species establishment, analyzing and visualizing potential ranges considering climate variables and other indicators at regional and landscape levels. This is particularly crucial as it helps fill gaps in knowledge that persist in assessing spatial scales of invasions and risks within countries. Interdisciplinary assessments at ecosystem and landscape levels provide more informative and effective strategies for future management and mitigating the impacts of environmental degradation caused by the establishment of invasive species.

Climate modeling of *R. japonica* distribution has been conducted in isolated studies^11^, ^14^, ^42^ over relatively small geographic areas. However, the dispersal pathways and habitat types most frequently colonized under different environmental conditions remain insufficiently studied. There is still a lack of understanding regarding the key factors and mechanisms driving the spread of species within this genus, including the influence of climate change, urbanization, aridification, and excessive moisture. It is essential to identify areas where the risk of expansion is particularly high or where the invasion is just beginning to determine the conditions that promote species spread. Future research should focus on predicting the potential ranges of other alien species, assessing their invasiveness and dispersal potential, and understanding interspecies interactions. This is crucial for gaining insight into ecosystem restructuring processes resulting from aridification and climate change.

In future research, it is crucial to study the risks of moving in during landscape transformations due to wartime activities, taxonomic and genetic characteristics, and species hybridization within the genus *Reynoutria*. It is necessary to investigate the distribution of the taxon, the mechanisms of its resilience in relation to urbanization, the urban heat island effect, moisture levels, and waterlogging.

## 5 CONCLUSIONS

For the first time, we conducted mapping, climate modeling, distribution forecasting under climate change scenarios, and risk assessment of *R. japonica* invasion. We obtained important results on the impact of extreme temperatures and moisture levels on the range expansion across 14 European countries. A projection of *R. japonica* expansion was carried out under two climate change scenarios up to the year 2100.

Based on 19 170 records of *R. japonica*, it has been demonstrated that Europe is suitable for the establishment of this taxon, including mountainous areas. The most critical factors limiting its distribution are the amount of precipitation during the hottest period of the year and the amplitude of temperature fluctuations throughout seasons and years, which are crucial for the species' survival. Temperature variability throughout the year significantly influences the species distribution. The average temperature of the coldest quarter will become increasingly significant with climate change by 2100.

In our modeling results, the expansion into northern regions of its range under a positive scenario will account for 13.6%, while under a negative scenario, it will reach 17.0%. However, there is projected narrowing of its range in southern regions due to increased temperatures, which constitutes 26.5% for both scenarios. A real forecasted contraction of the range by 9–13% is expected by 2100 in southern regions where the species is currently prevalent, with a shift towards northern regions of Ukraine, Belarus, and partially mountainous areas.

## Supporting information


**Data S1:** Supporting Information.

## Data Availability

The data that supports the findings of this study are available in the supplementary material of this article.
