# Supplementary material for: The spread of the invasive species Reynoutria japonica Houtt. will both expand and contract with climate change: results of climate modelling for 14 European countries
Source: Pest Manag Sci. 2025 Mar 20;81(7):3642–53. doi: 10.1002/ps.8732 (PMC12159380; doi:10.1002/ps.8732)
Supplement: Supplementary file 1 — Data S1: Supporting Information. [file PS-81-3642-s001.docx]

Table S1. Locations of *Reynoutria japonica* Houtt. identified in 2023-2024

(Authorship of finds - Dr. Miroshnyk N.)

| № | Location | Coordinates in decimal system | | Date of finding (year-month-day) |
| --- | --- | --- | --- | --- |
|  |  | latitude | longitude |  |
| **Ukraine** | | | | |
| 1 | Chernihiv, Gagarina St. 5, in the front garden near a 5-story building | 51.499972 | 31.258325 | 2023-08 |
| 2 | Chernihiv, in a flowerbed, bush + 8 sprouts, flowerbed with irises and quinoa, not watered or mowed | 51.5096334633073 | 31.329797862522682 | 2023-08-04 |
| 3 | Kyiv, Svyatoshynskyi district, in a flowerbed, Heroiv Kosmosu St. 15A | 50.438513 | 30.383338 | 2023-08-25 |
| 4 | Skvyra, Kyiv region, outskirts of the organic field of the Skvyra research station | 49.698689 | 29.675826 | 2023-07 |
| **Germany** | | | | |
| 5 | Large Tiergarten, Berlin city center, 10785 Berlin; on the shore of a reservoir, grows close to the water | 52.512765 | 13.356635 | 2023-07 |
| 6 | Heidestraße, 10557 Berlin | 52.532767 | 13.366227 | 2023-07 |
| 7 | Bellin, Ueckermunde (in a pine forest 200 m to the beach, littered roadside) | 53.7256210878326 | 14.035464048759488 | 2023-07-22 |
| 8 | Strandweg 14h, 17373 Ueckermünde, near the road, 300 m to the sea, on the outskirts of the pine forest | 53.736727, | 14.134033 | 2023-07 |
| 9 | 17373 Ueckermünde, Germany (1.5 m to the road) | 53.737570, | 14.134046 | 2023-07 |
| 10 | Intersection near outskirts of Ueckermünde | 53.733626 | 14.052965 | 2023-07 |
| 11 | Intersection near outskirts of Ueckermünde | 53.733626 | 14.052965 | 2023-07 |
| 12 | Chausseestraße 76, 17373 Ueckermünde, Germany, Ueckermünde Zoo, near the pedestrian path and water body | 53.732041 | 14.028074 |  |
| 13 | Ueckermünde, near the parking lot, near *Acer pseudoplatanus* L. | 53.732011 | 14.026135 |  |
| 14 | Ueckermünde, near the railway track, on the roadside in the bushes | 53.7335988 | 14.0544856 | 2023-07–28 |
| 15 | Ueckermünde Zoo, this is the yard of some estate | 53.732050 | 14.026133 | 2023-07–28 |
| 16 | Rathauspark Reinickendorf, Berlin | 52.590455166772486 | 13.323713443299582 | 2023-09-09 |
| 17 | Das Schmöckwitz, Wernsdorfer Strasse 43, 12527 Berlin, mature flowering, 10 individuals along the bridge and asphalt | 52.39257814003287 | 13.628990154811301 | 2023-09-14 |
| 18 | Das Schmöckwitz, Wernsdorfer Strasse 43, 12527 Berlin, in a deciduous forest on a stump | 52.3789488 | 13.6879225 | 2023-09 |
| 19 | Potsdam, blooming near the entrance to Lidl | 52.407824 | 13.075285 | 2023-08 |
| 20 | Potsdam, Schilfhof 28, roadside, blooming near the school | 52.381934 | 13.093282 | 2023-08 |
| 21 | District of Struppen, Saxon Switzerland National Park, roadside S 168 | 50.93166944640718 | 14.020553040473875 | 2023-08 |
| 22 | Gutenbergstraße 50, 14467 Potsdam, several individuals near asphalt and playground, very little land, almost all asphalted, near the road and house, no other vegetation | 52.402257909543366 | 13.067139725234984 | 2023-08 |
| 23 | Bad Schandau, along the road and forest near the reservoir, no other vegetation near the road and house | 50.923036 | 14.142515 | 2023-10-15 |
| 24 | Hauptstrasse 102, 01796 Struppen, Germany, near the bridge in the water | 50.933620 | 14.015065 | 2023-10-15 |
| 25 | Saxon Switzerland resort Rathen, outskirts of Rathen, near the Bastei bridge, roadside, ruderal habitat near the forest, large thickets of about 100 individuals, 50 m away, more thickets stretching for 30 m, over 300 plants in the lower valley | 50.958207 | 14.087610 | 2023-10-15 |
| 26 | Saxon Switzerland resort Rathen | 50.955751 | 14.089961 | 2023-10-15 |
| 27 | Brandenburg, near the main station | 52.406768 | 12.557401 | 2023-09-17 |
| 28 | Allee der Kosmonauten, Lichtenberg, Berlin, in a hedge | 52.514613 | 13.515203 | 2023-09-18 |
| 29 | Potsdam, Wublitzstraße 20, Reynoutria on the roadside, 1 m from the roadside | 52.432074 | 12.957744 | 2023-10-18 |
| 30 | Pietschkerstr. 14-16, Potsdam 14480, thickets near the dormitory - in the yard near the wall, on the border with the asphalted path | 52.377760220991725, | 13.128122097623823 | 2024-04 |
| 31 | Quedlinburg, near the main station, on the riverbank, halfway in the water, height up to 13 m, 7 individuals | 51.78560721268895 | 11.151465022212955 | 2024-05-09 |
| 32 | Skver on the territory of The Research Institute for Sustainability (Potsdam), height up to 17 m, 30 stems forming solid thickets in 4 places in different parts of the park | 52.406342, | 13.072741 | 2024-1-06 |
| 33 | Near tram tracks in garden plot area, height 1.7 m, 20 specimens | 52.3758010 | 13.0891773 | 2024-06-6 |
| 34 | Hybrid on garden plot area, height 3.5 m, 20 specimens, specifically not being destroyed | 52.378974 | 13.086847 | 2024-06-6 |
| 35 | Schlaatz, 14478 Potsdam. Near railway tracks, in forest belt, 10 plants, height up to 1.2 m | 52.375988 | 13.088562 | 2024-06-6 |
| 36 | Wasteland near tram tracks, 4 stems, height 1.3 m | 52.372981 | 13.101392 | 2024-06-6 |
| 37 | Babelsberg Park, Potsdam. 300 m from paved road and 20 m from sidewalk. Area approximately 100 m^2^. 500 specimens, height 0.3 m to 2.8 m, colony, 30 m to artificial canal. Near canal in waterfront zone, over 200 specimens, height up to 2 m, area 100 m^2^ | 52.410725 | 13.099144 | 2024-07-6 |
| 38 | Babelsberg Park, Potsdam. Canal shoreline 200 m^2^, 100 specimens, height up to 80 cm, under canopy of park trees, 80 m to water | 52.408939 | 13.095356 | 2024-07-6 |
| 39 | Karstadt Hbf - near railway tracks, monoculture, 200 plants, height 2.3 m | 53.159167 | 11.744048 | 2024-08-6 |
| 40 | Breddin Hbf, near railway tracks, colony 60 specimens, height up to 1.30 m | 52.886164 | 12.223881 | 2024-08-6 |
| **Hungary** | | | | |
| 41 | Beginning of Margit Island, Budapest, 500 m from water, not associated with water, near asphalt, 26 stems, height 1.8 m | 47.518016 | 19.043968 | 2024-05-06 |
| 42 | 500 m further into Margit Island, Budapest, from the first finding, 4 stems in the green zone near asphalt far from water, up to 60 cm tall (cut and regrowing) | 47.524334 | 19.047487 | 2024-05-06 |
| 43 | Margit Island, Budapest, 15 stems, height up to 40 cm, on the verge with asphalt, ruderal growth, on a hill, 100 m to water, not associated with water at all, all asphalted and gravel-filled | 47.534686 | 19.053409 | 2024-05-06 |
| 44 | Margit Island, Budapest, in flower beds - 130 specimens | 47.531754 | 19.051562 | 2024-05-06 |
| 45 | Margit Island, Budapest, all up to 20 cm tall and growing through ivy in shaded place, not connected with water, under a closed canopy of London plane and ash trees, 23 pieces, in the shade zone of Field Maple | 47.529682 | 19.050145 | 2024-05-06 |
| **Croatia** | | | | |
| 46 | Zagreb, Croatia, near the main entrance of the Faculty of Forestry and Wood Technology, University of Zagreb. Height up to 2 m, in the middle of the hedge, 25 specimens. | 45.82434326174932 | 16.029274317793256 | 2024-05-23 |

**Table S2**. Initial environmental variables utilized to model the distribution of Reynoutria japonica Houtt.

| Code | Environmental variable | Unit |
| --- | --- | --- |
| Bio1 | Annual average temperature | °C |
| Bio2 | Mean diurnal range | °C |
| Bio3 | Isothermality (ВІО 1/ BIO 7) х 100 | % |
| Bio4 | Seasonality of temperature (coefficient of variation) | °C |
| Bio5 | Highest temperature of the hottest month | °C |
| Bio6 | Lowest temperature of the coldest month | °C |
| Bio7 | Annual temperature variation (BIO 5 – BIO 6) | °C |
| Bio8 | Average temperature of the rainy quarter months | °C |
| Bio9 | Average temperature of the driest quarter months | °C |
| Bio10 | Average temperature of the hottest quarter months | °C |
| Bio11 | Average temperature of the coldest quarter months | °C |
| Bio12 | Annual precipitation | mm |
| Bio13 | Precipitation of the rainiest month | mm |
| Bio14 | Precipitation of the driest month | mm |
| Bio15 | Precipitation seasonality (coefficient of variation) | mm |
| Bio16 | Precipitation of the rainiest quarter months | mm |
| Bio17 | Precipitation of the driest quarter months | mm |
| Bio18 | Precipitation of the hottest quarter months | mm |
| Bio19 | Precipitation of the coldest quarter months | mm |

Table S3. Climate change scenarios according to the GISS-E2-1-G model (Ma et al., 2022)

| Scenario name and code | Years of projection | Global temperature characteristics | Description of processes |
| --- | --- | --- | --- |
| ssp126 | 2041 – 2060 | global temperature increase by 1.75 °C | The most optimistic climate change scenario is ssp 126, which assumes that humanity will follow a sustainable development path, reduce greenhouse gas emissions and adapt to climate change. The global average temperature will rise by 1.5°C compared to pre-industrial levels in 2024 (1.34°C) and peak at 1.7°C in 2050, before starting to decline to 1.4°C in 2100 |
| ssp126 | 2081 – 2100 | increase by 2,97 ̊С |  |
| ssp585 | 2041 – 2060 | increase by 2,06 ̊С | The most pessimistic scenario is ssp 585, with high greenhouse gas emissions, assumes that humanity will continue to grow economically and consume resources without concern for the environment and equity. The global average temperature will increase by 2.5°C compared to pre-industrial levels, rising by 5.03°C in 2057 and continuing to rise to 5.2°C in 2100. |
| ssp585 | 2081 – 2100 | increase by 5,62 ̊С |  |


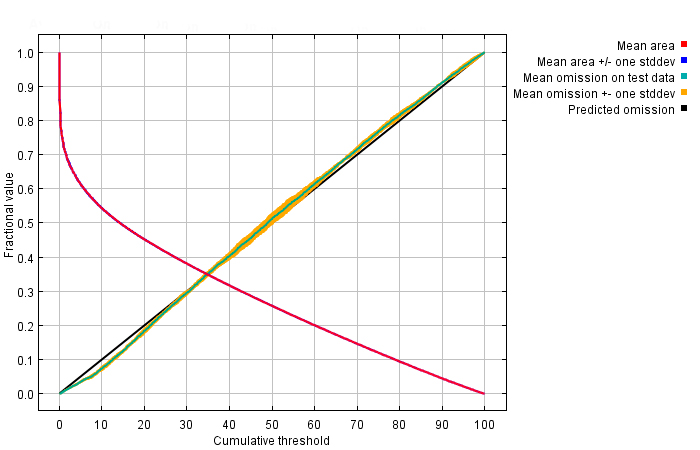


Figure S 1. Analysis of omission and predicted area (AUC) on the cumulative threshold for test observations for *R. japonica*


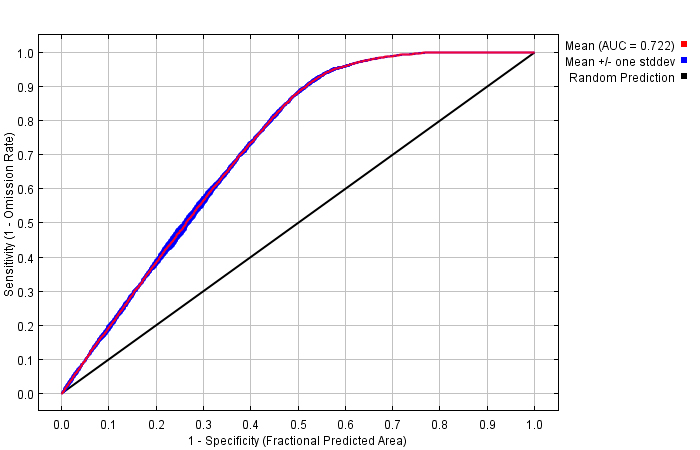


Figure S 2. ROC (Receiver Operating Characteristic) curve for test observations of *R. japonica*, averaged across four runs and model cross-validation using jackknife method, where the area under this curve (AUC) for test data is 0.724; random prediction (AUC = 0.5)

Table S4 . Contribution of each variable to the MaxEnt model of *R. japonica* distribution

| Variable | Percent contribution | Permutation importance |
| --- | --- | --- |
| **BIO 4** | **38.6** | **11.4** |
| **BIO 9** | **23,6** | **8,9** |
| **BIO 3** | **22,4** | **14,4** |
| **BIO 10** | **6.1** | **5.5** |
| **BIO 11** | **4,5** | **7,8** |
| **BIO 18** | **1,9** | **27,6** |
| BIO 7 | 1.2 | 3,8 |
| BIO 8 | 0.6 | 0,4 |
| BIO 1 | 0.4 | 1,4 |
| BIO 12 | 0,3 | 9.8 |
| BIO 5 | 0.2 | 2.7 |
| BIO 15 | 0.2 | 6.1 |
| BIO 19 | 0,1 | 0.1 |
| BIO 2 | 0 | 0.1 |
| BIO 13, 17, 6, 14, 16 | 0 | 0 |

Note. The most important variables are highlighted in bold.

Table S5. Contribution of the most important variables to the MaxEnt model of *R. japonica* distribution after 4 model runs

| Variable | Percent contribution | Permutation importance |
| --- | --- | --- |
| **BIO 4** | **39.2** | **11.9** |
| **BIO 3** | **22.4** | **14.5** |
| **BIO 9** | **21.9** | **9.1** |
| **BIO 10** | **5** | **4.8** |
| **BIO 18** | **4.4** | **27.7** |
| BIO 11 | 2.2 | 7.4 |
| BIO 6 | 1.1 | 0 |
| BIO 1 | 1.1 | 1.8 |
| BIO 2 | 0.8 | 0.1 |
| **BIO 7** | **0.7** | **4.8** |
| BIO 8 | 0.6 | 0.5 |

Note. The most important variables are highlighted in bold.


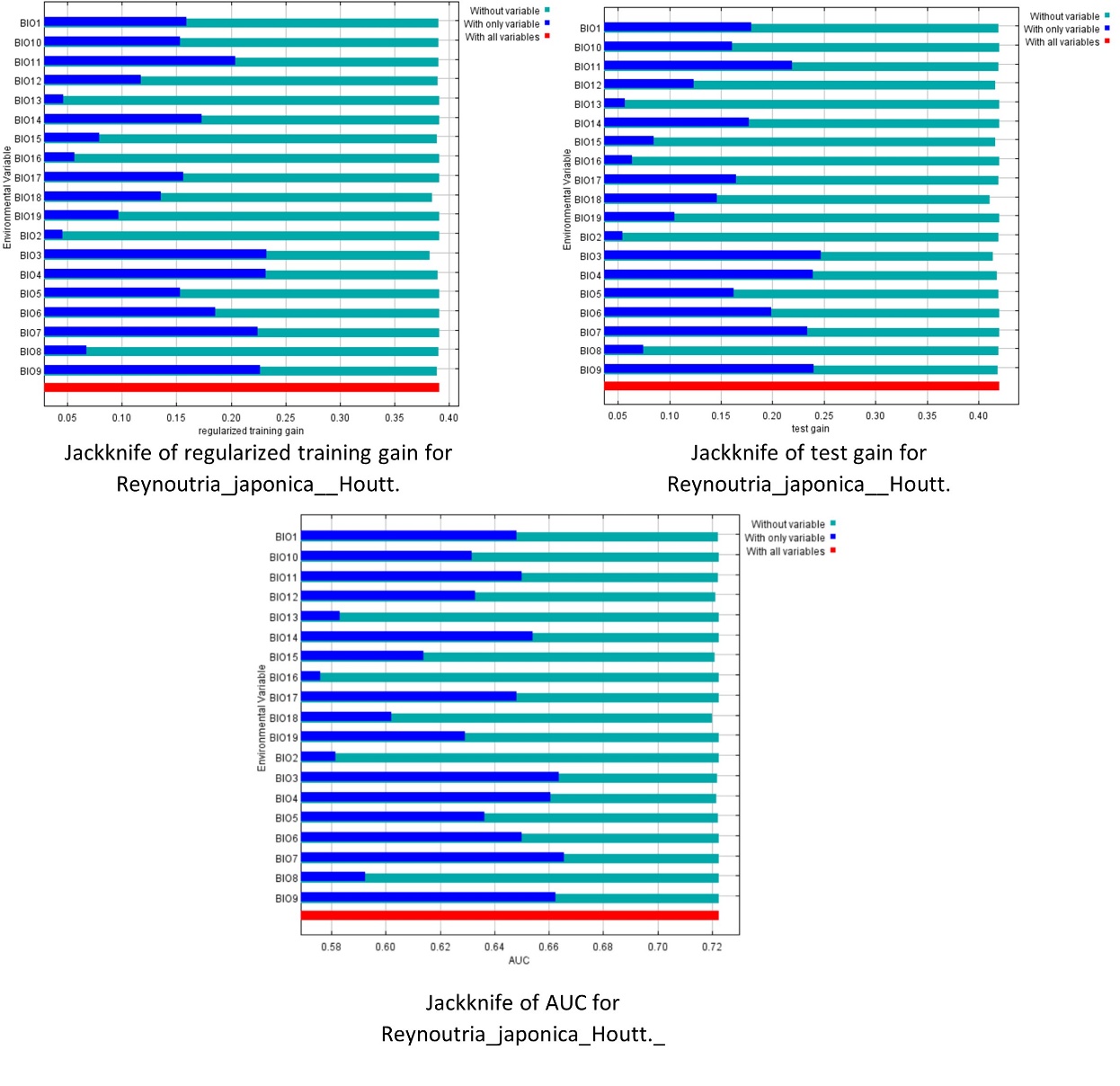


Figure S 3. Testing the model for the distribution of *R. japonica* using the Jackknife tool
